# Primary sudden onset severe headache: Prognosis and factors influencing it

**DOI:** 10.1186/1129-2377-14-S1-P133

**Published:** 2013-02-21

**Authors:** BK Kim

**Affiliations:** 1 Eulji Hospital, Korea, Republic of

## Introduction

Sudden onset severe headache, so called thunderclap headache, is a common condition that sometimes indicates a life-threatening conditions such as subarachnoid haemorrhage (SAH). Little is known about the prognosis of primary sudden onset severe headache (PSOH). Objectives To investigate the recurrence rate of sudden onset severe headache with normal brain imaging and lumbar puncture and factors associated with its recurrence.

## Methods

We have performed a prospective study of 80 consecutive patients with PSOH. PSOH was defined as reaching maximum intensity (visual analogue scale ≥7) within a few seconds with normal CSF and brain imaging. We investigate recurrence rate of PSOH and prognostic factors associated with its recurrence: sex, age, provoking factors, etc.

## Results

Mean age was 45 years (15-70) and female was 62%. Mean follow up duration was 504 days (SD=272 days, ranged from 2 to 30 months) from first visit. 43 patients with PSOH experienced at least two attacks. Recurrence of PSOH after 1st visit occurred in 15 cases. Nine out of 15 patients experienced recurrence within two weeks. Six patients experienced recurrence three month after first visit. All factors examined including age, sex, provoking factors, severity of headache, number of attacks at first visit, history of other primary headache and presence of reversible cerebral vasoconstriction syndrome were not associated with recurrence of PSOH.

## Conclusion

We observed that the recurrence of PSOH after one month is not common. Recurrence usually occurred within 2 weeks after first visit.
